# Timing of initial symptom onset during milk and wheat challenges: A retrospective study

**DOI:** 10.1002/iid3.1174

**Published:** 2024-02-02

**Authors:** Noriyuki Yanagida, Takanori Minoura, Sakura Sato, Kyohei Takahashi, Ken‐ichi Nagakura, Kiyotake Ogura, Takaaki Itonaga, Yoko Miura, Naoko Fusayasu, Motohiro Ebisawa

**Affiliations:** ^1^ Department of Allergy, Clinical Research Center for Allergy and Rheumatology NHO Sagamihara National Hospital Kanagawa Japan; ^2^ Department of Pediatrics NHO Sendai Medical Center Sendai Japan

**Keywords:** child, food allergy, initial symptom, milk, oral food challenge, time, wheat

## Abstract

**Background:**

Allergic reactions to milk appear sooner than those to hen's eggs, irrespective of the total dose of the oral food challenges (OFCs) and type of matrices. The reported median times for the first symptom occurrence are 20–30 min with milk and 50–60 min with eggs. However, allergic reactions due to wheat have not yet been fully investigated.

**Methods:**

This study retrospectively collected data from OFC for milk and wheat conducted at Sagamihara National Hospital and Sendai Medical Center from 2009 to 2023. The time from the start of the OFC to the onset of symptoms was compared between children with cow's milk and wheat allergy.

**Results:**

Twenty‐five and 13 children reacted to single‐dose OFCs with milk products equivalent to 25 mL of raw cow's milk or 15 g of udon noodles, respectively. The median ages of patients with positive challenges were 1.4 and 2.8 years for milk and wheat, respectively, and the median times for the first symptom occurrence were 20 min and 53 min, respectively (*p* = .006).

**Conclusion:**

This multicenter study was the first to examine the time of symptom appearance during single‐medium‐dose milk and wheat challenges. Allergic reactions to wheat appear later than those for milk during OFC. For multiadministration OFC for wheat, the dosing interval should be longer than 60 min. Our findings can help improve the safety of OFCs.

## Graphical abstract

Allergic reactions to wheat appear later than milk during oral food challenge. For oral food challenge (OFC) for wheat, the dosing interval should be longer than 60 min.

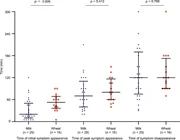

## 1 INTRODUCTION

Anaphylaxis and severe symptoms are frequently observed during milk and wheat oral food challenges (OFCs).^1^ Allergic reactions to milk are more rapid in onset and resolution than those to eggs, independent of the total dose of the OFC and the type of matrix.^2^, ^3^ The reported median times to first symptom onset following single‐dose milk and egg OFCs are 20–30 and 50–60 min, respectively.^2^, ^3^ However, allergic reactions to wheat have not been fully investigated.^4^ We conducted a multicenter retrospective study to clarify the timing of symptom onset in single‐dose wheat OFC.

## 2 METHODS

### 2.1 Study design

This study is a multicenter retrospective study, analyzing the results of single‐medium‐dose milk and wheat OFCs conducted at Sagamihara National Hospital and Sendai Medical Center from January 2010 to March 2023.

### 2.2 Oral food challenge

OFCs were administered according to the Japanese Guidelines for Food Allergy 2020,^5^ for both cow milk (850 mg, equivalent to 25 mL milk) and wheat (390 mg, equivalent to 15 g of udon noodle) for the first time (Table 1). For the milk OFC, skimmed milk powder (Milkona®) (Tamakona®) or lactic beverage (Yakult®) (Yakult®) was used. The evaluation method for symptom severity is shown in Table 2. OFC was considered positive only if objective clinical reactions were observed in this study. The challenge food was not divided, but rather administered to all participants simultaneously. The times of initial and peak symptom appearance and disappearance were recorded during the OFC, and symptoms were observed for at least 2 h after the participants consumed the challenge foods. After obtaining a negative result for the milk OFC, patients were allowed to consume almost all products containing milk except milk and yogurt. After obtaining a negative result for the wheat OFC, patients were allowed to consume almost all products containing wheat, except for large amounts of bread or noodles.

**Table 1:** Single‐dose oral food challenge with milk and wheat proteins detected in the challenge foods.

Challenge food	Milk/wheat/protein used (mg)	Origin of material
Single‐dose milk OFC	850	Milk drink contains cooked skim milk powder heated at 125°C for 30 s and spray‐dried for 3 s, hydrogenated maltose starch, fragrance, and 100 mL of water (Milkona®) (Tamakona®).
		Commercially available lactic beverage (Yakult®) (Yakult®)
Single‐dose wheat OFC	390	15 g of boiled udon noodles

*Note*: The challenge foods were prepared in a nutrition management room. In the OFC, the challenge foods were administered simultaneously.

Abbreviation: OFC, oral food challenge.

**Table 2:** Severity of symptoms during the oral food challenge.

Grade	1 (mild)	2 (moderate)	3 (severe)
Skin	Localized urticaria, exanthema, wheal, pruritus	Generalized urticaria, exanthema, wheal, pruritus	‐
	Swollen eyelid or lip	Swollen face	‐
Gastrointestinal tract	Pruritus of the throat or oral cavity	Throat pain	‐
	Mild abdominal pain	Moderate abdominal pain	Cramps
	Nausea, emesis, diarrhea	Recurrent emesis, diarrhea	Continuous emesis, loss of bowel control
Respiratory tract	Intermittent cough, nasal congestion, sneezing, rhinorrhea	Repetitive cough	Persistent cough, hoarseness, “barking” cough
	‐	Chest tightness and wheezing detectable via auscultation	Audible wheezing, dyspnea, cyanosis, saturation <92%, swallowing or speaking difficulties, throat tightness, respiratory arrest
Cardiovascular	‐	Pale face, mild hypotension, tachycardia (increase >15 beats/min)	Hypotension, dysrhythmia, severe bradycardia, cardiac arrest
Neurological	Change in activity level, tiredness	Light‐headedness, feeling of “pending doom,” somnolence, headache	Confusion, loss of consciousness, incontinence

*Note*: The severity score is based on the organ system most affected by the symptoms. Hypotension was defined as systolic blood pressure <70 mmHg for children aged 1 month to 1 year, <70 + (2 × age) mmHg for children aged 1–10 years, and <90 mmHg for children aged 11 years and above. Mild hypotension was defined as systolic blood pressure <80 mmHg for children aged 1 month to 1 year, <80 + (2 × age) mmHg for children aged 1–10 years, and <100 mmHg for children aged 11 years and above. Wheezing detected by stethoscopic auscultation was defined as mild wheezing. Audible wheezing was defined as wheezing detected without a stethoscope. The severity score is defined in the anaphylaxis guidelines for Japan.

### 2.3 ImmunoCAP analysis

Serum levels of specific immunoglobulin E (sIgE) against milk, casein, wheat, and omega 5 gliadin (Immuno CAP™; Thermo Fisher Scientific/Phadia) were measured within 6 months of OFC. If the sIgE level for the causative foods was >100 kU_A_/L, a dilution measurement was performed.

### 2.4 Eligibility criteria

Children who were sensitized to milk or wheat allergies received OFC. Here, “sensitization” was defined as the levels of sIgE to milk and wheat of >0.1 kU_A_/L. Children with non‐IgE‐mediated food allergies, lactose intolerance, or congenital lactase deficiency were excluded. Children with objective symptoms during OFC, as well as those with high antigen‐specific IgE responses to causative foods, were also enrolled.

### 2.5 Statistical methods

Data are expressed as medians, ranges, or interquartile ranges. The Mann–Whitney *U* test was used, and a *p* < .05 was considered statistically significant. All analyses were performed using SPSS software (version 24.0; SPSS Inc.).

### 2.6 Sample size calculation

Sample size was calculated by G*Power 3.1.9.4. We compared the results of this study with those of a previous study.^2^ We assumed that the times of symptom appearance would be 30 ± 60 (milk) and 60 ± 60 min (wheat). We hypothesized that the ratio of the number of milk OFC‐reactive patients to the number of wheat OFC‐reactive patients would be 2:1 according to the prevalence of the allergy in Japan.^5^ Power was 0.8, and alpha was .05 for both OFCs. The optimal sample size was more than 25 (milk) and 13 (wheat).

### 2.7 Ethical considerations

Written informed consent was obtained from all parents or guardians. If capable, we obtained consent from children of 10 years or older. All data were anonymized before the analysis. This study was approved by the Ethics Committee of the Sagamihara National Hospital (Approval number: 2014‐3‐18).

## 3 RESULT

### 3.1 Enrollment

**Figure 1:**
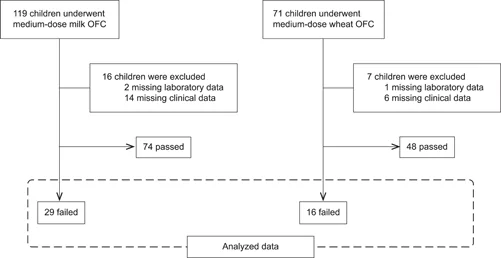
Patient enrollment. A single medium‐dose (OFC) (Table 1) was administered. None of the children consumed extensively heated forms of milk or wheat before the OFC. OFC, oral food challenge.

Of 190 children, 23 did not meet our eligibility criteria. We further excluded 122 children who passed the milk/wheat OFC, and 45 children who failed were finally analyzed (Figure 1). All children were confirmed to consume low‐dose milk or wheat safely by low‐dose OFC before single‐medium‐dose OFC. Every child underwent either a milk or wheat OFC. The median ages of the children were 1.4 (1.0–4.1) years and 2.8 (1.9–5.3) years for milk and wheat OFCs, respectively (Table 3). The median serum‐specific immunoglobulin E (sIgE) levels for milk and wheat were 13.2 and 23.3 kU_A_/L, respectively.

**Table 3:** Background of patients who received single‐dose OFC.

	Single‐dose milk OFC (*n* = 29)	Single‐dose wheat OFC (*n* = 16)
Sex (male)	20 (69%)	14 (88%)
Age (years), median	1.4 (1.0–4.1)	2.8 (1.9–5.3)
History of immediate reaction to causative food	22 (76%)	12 (75%)
History of anaphylaxis to causative food	10 (35%)	7 (44%)
Other food allergies	27 (93%)	15 (94%)
Atopic dermatitis, current	22 (76%)	11 (69%)
Bronchial asthma, current	5 (17%)	3 (19%)
Allergic rhinitis, current	1 (3%)	2 (13%)
Milk sIgE (kU_A_/L)	13.2 (4.1–47.2)	
Casein sIgE (kU_A_/L)	5.5 (3.1–41)	
Wheat sIgE (kU_A_/L)		23.3 (9.0–30.1)
Omega 5 gliadin sIgE (kU_A_/L)		1.2 (0.7–4.0)
Total IgE (IU/mL)	426 (192–1240)	450 (179–1256)

*Note*: Data are expressed as *n* (%) or median values, with 25%–75% interquartile ranges provided in parentheses. History of immediate reaction to causative food was recorded before the OFC. All patients reacted during the OFC. No patient could tolerate “extensively heated” forms of milk and wheat before the challenge. Atopic dermatitis was well‐controlled in the study population.

Abbreviations: OFC, oral food challenge; sIgE, specific immunoglobulin E.

### 3.2 Severity of symptoms

Of the 45 children, 25 (86%) and 13 (81%) exhibited moderate symptoms of milk and wheat allergies, respectively (Table 4). No patient had severe reactions.

**Table 4:** Data related to the severity of reactions induced during a positive challenge and subsequent treatment.

Organ system affected	Single‐dose milk OFC (*n* = 29)	Single‐dose wheat OFC (*n* = 16)	*p* value
Skin	25 (86%)	15 (94%)	.641
Time of initial symptom appearance (min)	43 (25–59)	65 (48–75)	.011
Time of peak symptom appearance (min)	75 (60–115)	80 (60–113)	.940
Time of symptom disappearance (min)	125 (91–195)	120 (90–180)	.800
Respiratory	12 (41%)	6 (38%)	>.999
Time of initial symptom appearance (min)	20 (13–30)	63 (42–71)	<.001
Time of peak symptom appearance (min)	35 (28–53)	70 (48–79)	.003
Time of symptom disappearance (min)	60 (45–78)	90 (70–120)	.005
Gastrointestinal	7 (24%)	4 (25%)	>.999
Time of initial symptom appearance (min)	34 (20–203)	60 (30–83)	.164
Time of peak symptom appearance (min)	62 (20–225)	90 (60–113)	.429
Time of symptom disappearance (min)	105 (42–278)	100 (80–118)	>.999
Neurological	0 (0%)	1 (6%)	.356
Time of initial symptom appearance (min)		70	
Time of peak symptom appearance (min)		80	
Time of symptom disappearance (min)		95	
Cardiovascular	0 (0%)	0 (0%)	>.999
Severity			>.999
Mild (Grade 1)	4 (14%)	3 (19%)	
Moderate (Grade 2)	25 (86%)	13 (81%)	
Severe (Grade 3)	0 (0%)	0 (0%)	
Treatment			
Antihistamine (oral)	23 (79%)	13 (81%)	>.999
Steroid (oral)	4 (20%)	6 (11%)	>.999
Fluid infusion (intravenous)	1 (3%)	0 (0%)	.356
β2 stimulant inhalation	9 (31%)	7 (44%)	.726
Adrenaline (intramuscular)	0 (0%)	0 (0%)	>.999
No treatment	4 (14%)	2 (13%)	>.999

*Note*: Data are presented as *n* (%) or medians, as appropriate. Symptom severity was defined based on the severity of symptoms in the most affected organ (Table 2).

### 3.3 Time of initial symptoms

**Figure 2:**
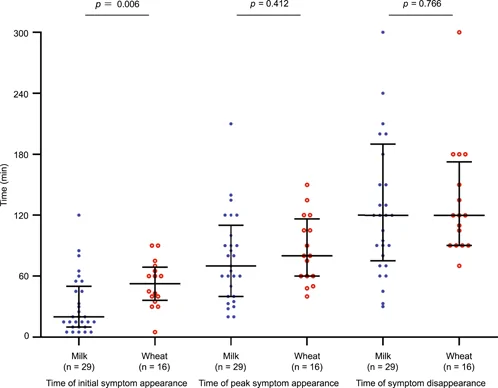
Minutes to symptom initiation, peak, and disappearance after single‐medium‐dose oral food challenges (OFCs). The times of initial symptom onset for milk and wheat are 28 (8–60) and 60 (28–90) min, respectively, in 10/16 (63%), 13/16 (81%), and 15/16 (94%) patients. Symptoms appeared within 30, 60, and 90 min in 20/53 (38%), 28/53 (53%), and 41/53 (77%) patients, respectively.

**Figure 3:**
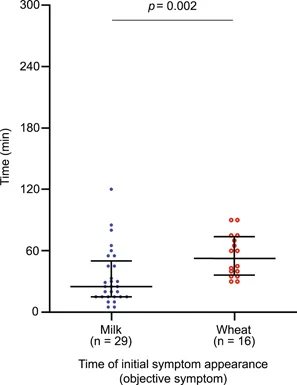
Time of initial symptom onset (objective symptom). The median times of the first objective symptom onset were 25 and 53 min (*p* = .042) in the milk and wheat oral food challenges, respectively.

The initial symptoms occurred at median times of 20 (10–50) and 53 (36–69) min for the milk and wheat OFCs, respectively (*p* = .006) (Figure 2). The median times to the onset of the first objective symptoms were 25 (15–50) and 53 (36–74) min for the milk and wheat OFCs, respectively (*p* = .002) (Figure 3). For these OFCs, the median times to the peak symptom onset were 70 (40–10) and 80 (60–116) min (*p* = .412), respectively, and the symptoms disappeared after 120 (75–190) and 120 (90–173) min, respectively (*p* = .766) (Figure 2). Positive symptoms were observed ≤30 min after ingestion in 66% (19/29) and 19% (3/16) of patients who underwent milk and wheat OFCs, respectively. Positive symptoms were observed >60 min after ingestion in 17% (5/29) and 31% (5/16) of patients who underwent the milk and wheat OFCs, respectively.

## 4 DISCUSSION

This multicenter study is the first to examine the time of symptom onset in single‐medium‐dose milk and wheat OFCs. Allergic reactions elicited by wheat were found to occur later than those elicited by milk, and previous studies have shown that allergic reactions induced by eggs appear later than those induced by milk.^2^, ^3^ Thus, careful attention should be paid to children for at least 1 h after administering wheat or egg OFCs. Moreover, our data include the onset time of mild subjective symptoms that do not halt OFC. The onset time of objective symptoms was found to be later than that of subjective symptoms. Considering this late onset time, a 1 h interval of OFCs should be justified for the safety of the challenge.

A single‐dose challenge would be ideal to determine the symptom‐provoking dose; however, it is practically difficult to determine the appropriate dose in terms of the safety of the dose. Therefore, if the challenge food is to be divided into several portions, the dosing interval should be lengthened. Longer intervals between doses reduce severe reactions in individuals undergoing wheat OFCs.^4^ Therefore, these OFCs can be safely administered to children at intervals of 30 min rather than 15 min.^4^ Kitamura et al. reported that wheat OFCs at 60‐min intervals are safer than those at 30‐min intervals.^6^ Late initial symptoms explain the reduced risk of adverse reactions with intervals longer than 30 min between doses, which may help in avoiding excessive intake. Indeed, in our study, 34% of the patients who underwent the milk OFC and 81% of those who underwent the wheat OFC reacted after 30 min. Moreover, positive symptoms were observed >60 min after ingestion in 17% and 31% of the patients who underwent the milk and wheat OFCs, respectively. Therefore, our study findings support longer intervals than 60 min for wheat OFC.

Data from 12 healthy volunteers showed that within 15 min after gluten ingestion, serum gliadin levels were elevated from baseline in all participants and peaked 15–90 min later.^7^ Among the volunteers, nine individuals (75%) showed peak levels 30 min or later after gluten ingestion without cofactor involvement. This late absorption at the peak level may explain why most initial symptoms in wheat OFC start later than 30 min after the start of the OFC. Baked milk OFC involving up to five doses administered every 10–20 min is associated with a higher requirement of adrenaline administration and frequent respiratory symptoms, with delayed reactions occurring ≥60 min after the start of the OFC.^8^ Short dosing intervals during OFC cause severe adverse reactions in children with milk allergy.^9^ Based on statistical calculations, among patients who received multiple doses of epinephrine, 95% had shorter‐than 30.3‐min dosing intervals, and OFCs with dosing intervals of 30 min or longer were recommended to improve the safety of milk OFC by reducing the incidence of severe anaphylactic adverse reactions.^9^ A quarter of patients with cow's milk allergy reported severe symptoms with OFC at 15‐min intervals.^10^ Moreover, the threshold dose and severity of symptoms are variable and unpredictable, even in a second OFC.^11^ Our study suggests that protocols for milk OFC should have a dosing interval of more than 30 min to improve the overall safety of the OFC. Physicians should carefully observe children undergoing wheat OFCs, similar to those undergoing egg OFCs, especially for 60 min after the initial wheat dose and for at least 2 h after the last wheat dose.^12^ Symptoms during milk OFCs may appear earlier than those during wheat OFCs, and the observation period may vary depending on the antigen. Further studies are needed to elucidate the mechanisms underlying the different timings of symptoms between milk and wheat.

**Figure 4:**
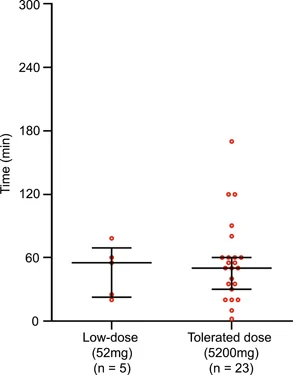
Minutes until initial symptom onset after low‐dose and full‐dose wheat oral food challenge (OFC). The median times until initial symptom onset in the low‐dose (*n* = 5) and full‐dose (*n* = 23) wheat OFC groups were 55 and 50 min, respectively.

Our study has several limitations. First, the OFCs were nonblinded and open. However, all patients with subjective symptoms exhibited objective symptoms, which appeared earlier in the milk OFC group than in the wheat OFC group. Second, although this was a multicenter study, the sample size was small, which was insufficient for multivariate analysis. Therefore, larger‐scale studies are warranted. Third, treatments for symptoms, such as antihistamines, steroids, and beta 2 agonist inhalations, may have influenced the peak and disappearance of symptoms. Fourth, the total target dose may have affected the time of initial symptoms. For the medium‐dose OFC, the protein doses for cow's milk and wheat were determined based on the doses known to improve the quality of life.^13^, ^14^ Although we could not perform a comparative analysis, using our limited data,^1^ we noted that the times of initial symptoms in the low‐dose (*n* = 5) and full‐dose (*n* = 23) wheat OFC groups were 55 and 50 min, respectively (Figure 4). Therefore, the total OFC dose had little effect. Lastly, wheat OFC is conducted using udon noodles, which are not always the universal wheat product in terms of protein concentration and gluten formation. This might affect the time interval of symptom appearance.

In conclusion, allergic reactions to milk occur earlier than those to wheat. The time between doses should be greater than 60 min for wheat OFCs. Our results provide evidence for the timing of initial symptoms for wheat OFCs, with existing evidence for egg and milk OFCs.

## AUTHOR CONTRIBUTIONS

Noriyuki Yanagida designed the study, wrote the manuscript, analyzed the data, and generated the figures. Takanori Minoura, Sakura Sato, Kyohei Takahashi, Ken‐ichi Nagakura, Kiyotake Ogura, Takaaki Itonaga, Yoko Miura, Naoko Fusayasu, and Motohiro Ebisawa revised the manuscript and critiqued the figures. Noriyuki Yanagida, Takanori Minoura, Sakura Sato, Kyohei Takahashi, Ken‐ichi Nagakura, Kiyotake Ogura, Takaaki Itonaga, Yoko Miura, Naoko Fusayasu, and Motohiro Ebisawa collected data. Kyohei Takahashi created a study database. Motohiro Ebisawa provided advice on the study design. All the authors have read and approved the final version of the manuscript.

## CONFLICT OF INTEREST STATEMENT

Motohiro Ebisawa received lecture fees from Viatris, Sanofi, and ARS Pharmaceuticals. All other authors declare no conflict of interest.

## ETHICS STATEMENT

This study was approved by the Ethics Committee of the Sagamihara National Hospital (Approval number: 2014‐3‐18).

## Data Availability

The data that support the findings of this study are available from the corresponding author upon reasonable request.
